# Phylogenetic Reconstruction and Functional Characterization of the Ancestral Nef Protein of Primate Lentiviruses

**DOI:** 10.1093/molbev/msad164

**Published:** 2023-07-18

**Authors:** Abayomi S Olabode, Mitchell J Mumby, Tristan A Wild, Laura Muñoz-Baena, Jimmy D Dikeakos, Art F Y Poon

**Affiliations:** Department of Pathology & Laboratory Medicine, Western University, London, Canada; Department of Microbiology & Immunology, Western University, London, Canada; Department of Microbiology & Immunology, Western University, London, Canada; Department of Microbiology & Immunology, Western University, London, Canada; Department of Microbiology & Immunology, Western University, London, Canada; Department of Pathology & Laboratory Medicine, Western University, London, Canada; Department of Microbiology & Immunology, Western University, London, Canada; Department of Computer Science, Western University, London, Canada

**Keywords:** ancestral reconstruction, human immunodeficiency virus, primate lentivirus, negative effector protein

## Abstract

Nef is an accessory protein unique to the primate HIV-1, HIV-2, and SIV lentiviruses. During infection, Nef functions by interacting with multiple host proteins within infected cells to evade the immune response and enhance virion infectivity. Notably, Nef can counter immune regulators such as CD4 and MHC-I, as well as the SERINC5 restriction factor in infected cells. In this study, we generated a posterior sample of time-scaled phylogenies relating SIV and HIV Nef sequences, followed by reconstruction of ancestral sequences at the root and internal nodes of the sampled trees up to the HIV-1 Group M ancestor. Upon expression of the ancestral primate lentivirus Nef protein within CD4${}^{+}$ HeLa cells, flow cytometry analysis revealed that the primate lentivirus Nef ancestor robustly downregulated cell-surface SERINC5, yet only partially downregulated CD4 from the cell surface. Further analysis revealed that the Nef-mediated CD4 downregulation ability evolved gradually, while Nef-mediated SERINC5 downregulation was recovered abruptly in the HIV-1/M ancestor. Overall, this study provides a framework to reconstruct ancestral viral proteins and enable the functional characterization of these proteins to delineate how functions could have changed throughout evolutionary history.

## Introduction

Human immunodeficiency virus type 1 (HIV-1) was introduced into human populations via multiple zoonotic transmission events of the simian immunodeficiency virus (SIV) infecting the common chimpanzee (*Pan troglodytes*), also referred to as SIVcpz (Hahn et al. 2000; Keele et al. 2006). These transmission events ultimately produced the four recognized groups of HIV-1 (denoted as M, N, O, and P), which phylogenetically cluster to different lineages of SIVcpz infecting the *P. t. troglodytes* subspecies (SIVcpzptt) (Gao et al. 1999; Keele et al. 2006; Sharp and Hahn 2011). Of these, HIV-1 group M (HIV-1/M) is responsible for the majority of infections within the human population (Sharp and Hahn 2011). Phylogenetic analyses have revealed that SIVcpz is a recombinant virus, where the $5^{\prime }$ region, *nef* and the $3^{\prime }$ long terminal repeat (LTR) region closely resembles SIV infecting the red-capped mangabey (*Cercocebus torquatus*, SIVrcm) (Bailes et al. 2003). The rest of the genome, which includes the *vpu*, *rev*, *tat*, and *env* genes, closely resembles SIV infecting several *Cercopithecus* species, including the greater-spot nosed monkey (*Cercopithecus nictitans*), mustached monkey (*Cercopithecus cephus*), and the mona monkey (*Cercopithecus mona*) (Bailes et al. 2003), henceforth denoted as the SIVgsn/mus/mon clade. As common chimpanzees predate upon primates such as those belonging to the *Cercocebus* and *Cercopithecus* species (Goodall 1986; Sharp and Hahn 2011), these predation events are believed to have led to the recombination of SIVrcm and SIVgsn/mus/mon within a superinfected chimpanzee host (Sharp and Hahn 2011).

The pathogenesis of SIVcpz infections within chimpanzees are similar to HIV-1 infections within humans (Keele et al. 2009; Etienne et al. 2011). Indeed, a progressive loss of CD4${}^{+}$ T cells and histopathological sequela consistent with end-stage acquired immunodeficiency syndrome (AIDS) observed in infected humans have been documented in infected wild chimpanzees (Keele et al. 2009). While the study of wild chimpanzees is challenging due to their endangered status (Fontsere et al. 2022), chimpanzee groups with a higher prevalence of SIVcpz infections exhibit significant population declines when compared to chimpanzee groups with lower SIVcpz prevalence (Rudicell et al. 2010). These findings suggest SIVcpz infections are associated with increases in chimpanzee morbidity and mortality in the wild. Conversely, the majority of African Old-World monkeys, such as SIV-infected sooty mangabeys (*Cercocebus atys*) and African green monkeys (*Chlorocebus sabaeus*), generally experience mostly nonpathogenic SIV infections (Silvestri 2009), with minimal progression to immune dysfunction and AIDS, despite often possessing high viral loads (Paiardini et al. 2009). Thus, the zoonotic transmission of SIV from Old-World monkeys to Great Apes contributed to the emergence of highly pathogenic SIV in the novel hosts.

The accessory protein Nef is unique to primate lentiviruses (Gifford 2012) and plays a significant role during viral pathogenesis (Kirchhoff et al. 2008). As with other HIV accessory proteins, Nef lacks enzymatic activity (Foster and Garcia 2008), and instead functions by directly interacting with an array of host proteins to commandeer cellular pathways/functions within infected cells (Fackler and Baur 2002; Michel et al. 2006; Jacob et al. 2021). A well-described function of Nef proteins derived from SIV and HIV-1 lineages is the ability to downregulate the CD4 entry receptor from the surface of infected cells. This function is mediated by a dileucine motif located within the disordered C-terminal region of Nef (Greenberg et al. 1998). Nef-mediated downregulation of cell-surface CD4 reduces the rate of superinfection and also limits the antibody-dependent cell-mediated cytotoxicity (ADCC) response, where Envelope (Env) in the CD4-bound conformation is preferentially targeted by ADCC-mediating antibodies (Richard et al. 2015). Furthermore, Nef enhances virion infectivity by targeting the cell-surface serine incorporator 5 (SERINC5) restriction factor, and to a lesser extent SERINC3 (Rosa et al. 2015). In the absence of Nef, SERINC5 is readily incorporated into the outer viral membrane during egress (Dai et al. 2018; Mumby et al. 2021). Virus particles with incorporated SERINC5 cannot undergo fusion with the membrane of subsequent target cells, thereby inhibiting replication (Diehl et al. 2021). To overcome this restriction, Nef triggers the internalization of SERINC5 from the cell surface by bridging SERINC5 to the endolysosomal network via multiple conserved Nef functional motifs (Trautz et al. 2016; Shi et al. 2018; Mumby et al. 2021). Internalized SERINC5 is ultimately directed to the lysosome where it is degraded, thereby promoting the restoration of virion infectivity (Shi et al. 2018).

Interestingly, it was discovered that Nef is able to downregulate SERINC5 in a species-independent manner. For instance, some Nef proteins derived from highly divergent HIV and SIV strains downregulated a variety of primate/Great Ape SERINC5 orthologs (Heigele et al. 2016). Furthermore, this functional ability of Nef also strongly correlates with the prevalence of primate lentiviruses in their respective natural host species (Heigele et al. 2016). This association demonstrates that Nef-dependent enhancement of virion infectivity via SERINC5 antagonism can provide favorable conditions for efficient intraspecies viral transmission and population dissemination (Heigele et al. 2016). While Nef proteins derived from modern-day SIVcpz generally antagonize SERINC5 efficiently (Heigele et al. 2016), it remains unclear if this function was selected over the course of SIVcpz adaptation to common chimpanzees prior to zoonotic human transmission, or if this function is conserved across primate lentivirus lineages. Considering most studies examining SIV Nef function use modern-day sequences, limited information about this protein throughout primate lentivirus evolutionary history exists. Herein, we used ancestral reconstruction (Joy et al. 2016) to infer the ancestral primate lentivirus (PLV) *nef* sequence to all primate lentiviruses, as well as the ancestral lineages preceding the transmission of SIVcpz from chimpanzees to humans as HIV-1/M. To determine if reconstruction of the ancestral PLV Nef protein was successful, we evaluated whether the ability of ancestral PLV Nef could perform two functions preserved in both SIV and HIV-1 Nef proteins; cell-surface CD4 and SERINC5 downregulation, previously demonstrated with contemporary SIV and HIV-1 Nef proteins. The characterization of Nef ancestors involved in the phylogeny from the ancestral PLV to the HIV-1/M ancestor allowed for an estimation of how Nef-mediated cell-surface CD4 and SERINC5 downregulation may have evolved throughout evolutionary time.

## Methods

### Phylogenetic Analysis

Supplementary figure S1, Supplementary Material online shows a graphical summary of all bioinformatic and experimental methods used in this study. A total of n=34 lentiviral (SIV and HIV) Nef protein sequences were obtained from the Los Alamos National Laboratory (http://www.hiv.lanl.gov/) and Genbank (https://www.ncbi.nlm.nih.gov/genbank/) databases. Our sequence selection was guided by a prior phylogenetic study on the origin of SIV (Worobey et al. 2010), which incorporated geological information on the isolation of host species on Bioko island to calibrate the molecular clock. Although there are substantially more HIV and SIV *nef* sequences available, we limited our analysis to these 34 representative sequences for two reasons. First, we used Bayesian sampling to accommodate the uncertainty in reconstructing the deep evolutionary history of primate lentiviral *nef* sequences. The computing time required for this sampling method to converge to the true posterior distribution of trees grows exponentially with the number of sequences. Second, we found that the majority of published *nef* sequences are either incomplete, represent laboratory strains, or represent clonal sequences sampled from the same host individual, which confer little information on the divergence among host species. Our objective is to characterize a small number of “deep” common ancestors relating primate lentiviruses from different host species, for which the reference set is sufficient to reconstruct.

From these reference Nef sequences, we generated a multiple sequence alignment using the MAFFT (v7.271) alignment program (Katoh and Standley 2013). A maximum-likelihood tree was reconstructed using IQ-TREE (version 1.3.11.1) (Nguyen et al. 2015) and was used as a starting tree for a Bayesian sampling analysis. We used BEAST (version 1.10.4) (Drummond and Rambaut 2007) to sample time-scaled phylogenies from the posterior distribution. Based on a previous study (Worobey et al. 2010), we used a Yule tree prior, and the JTT (Jones et al. 1992) amino acid substitution model with rate variation among sites modeled by a gamma distribution with four rate categories, and an uncorrelated lognormal clock model. We calibrated the evolutionary rate by specifying a normally distributed prior (mean=76.794 thousands of years; standard deviation=26.0) on the time to the most recent common ancestor (TMRCA) (Worobey et al. 2010). Four replicate chains were run for $10^{8}$ steps. We discarded the first $10^{7}$ steps from each chain sample as burn-in (the initial period where the location of the sampling process is strongly influenced by its starting conditions) and then generated a maximum clade credibility (MCC) tree from the remainder using TreeAnnotator (version 1.10.4) (Drummond and Rambaut 2007). Next, we thinned the chain samples down to 10,000 trees each. We calculated the frequencies of internal nodes, where each node is identified by the labels of its descendant tips, across all 40,000 trees, and then determined the most frequent combination of nodes from the root to the common ancestor of HIV-1/M. Finally, we randomly selected 1,000 trees from the subset of n=4,353 trees that were consistent with this maximum-support trajectory comprising eight internal nodes.

### Ancestral Sequence Reconstruction

Using the 34 extant Nef sequences, we used Historian (version 0.1) (Holmes 2017) to reconstruct the ancestral amino acid sequences for all internal nodes of the MCC tree, and for each of the 1,000 trees in our random sample. We chose this ancestral reconstruction program because it incorporates models of indel evolution, which occur at a relatively high frequency in *nef*, particularly in the anchor region (supplementary fig. S2, Supplementary Material online). To validate the accuracy of reconstructing ancestral sequences with Historian, we simulated the evolution of Nef extant sequences along the MCC tree using INDELible (v1.0.3; supplementary fig. S3, Supplementary Material online) (Fletcher and Yang 2009). The simulation was seeded at the root with the Historian-reconstructed ancestral sequence at the root of this tree. Genetic variation in the simulated alignments was specified by the expected number of substitutions per codon in the entire tree (scaling factor, SF={1.0,2.5,5.0,and10.0}). We assumed a transition/transversion ratio of κ=8.0, and either a zero indel rate or a constant ratio of indel to codon substitution rates of 0.01. Indels lengths (*L*) were drawn from a Lavalette distribution, $f(L)=((M-L+1)/(ML){)}^{a}$, under varying parameter settings for the exponent, a={1.5, 2.0, 3.0, 5.0}, and maximum indel size, M={4, 6, 8, 10, 20, 50, 100, 200} codons. This resulted in a total of n=124 parameter combinations. Next, we repeated the previous analysis workflow to reconstruct ancestral sequences for 25 replicate simulations for a total of 3,300 tests. We calculated the number of nucleotide differences between the true initial sequence and the reconstructed sequence at the root. In addition, we calculated the amino acid entropy for each alignment using the R package Bios2cor (Taddese et al. 2021).

### Cell Culture

CD4${}^{+}$ HeLa cells (NIH AIDS Reagent Program) were maintained in Dulbecco’s modified Eagle’s medium (DMEM) containing 4 mM l-glutamine (Cytiva Life Sciences, Vancouver, BC), 4.5 g/l glucose (Cytiva Life Sciences) and supplemented with 10% fetal bovine serum (FBS—Wisent, St. Bruno, QC, Canada), and 1% penicillin and streptomycin (HyClone, Logan, UT). The cells were grown at 37 ${}^{\circ }$C in the presence of 5% CO${}_{2}$ and were subcultured in accordance with the manufacturer’s recommendations.

### DNA Constructs

The reconstructed SIV *nef* sequences of the SIV/HIV phylogeny were generated using the GeneArt Gene Synthesis service (ThermoFisher). To convert the reconstructed amino acid sequences to nucleotides, we tabulated codon frequencies in the extant sequences at each position of the alignment, and mapped each residue to the most frequent codon. The nucleotide sequence encoding SIVmac239 *nef* utilized in this study was obtained from the SIVmac239 SpX plasmid (ARP-12249), which was contributed by Dr Donald Desrosiers through the National Institutes of Health (NIH) HIV Reagent Program (Division of AIDS, National Institute of Allergy and Infectious Diseases). The primary Nef sequences utilized in this study have been described previously (Mumby et al. 2021).

SIV and HIV-1 *nef* sequences were PCR amplified using a forward primer harboring a $5^{\prime }$ EcoRI restriction site, and a reverse primer harboring a $3^{\prime }$ BamHI restriction site. To generate a plasmid expressing an Nef-eGFP fusion protein, the reverse primer was additionally designed to mutate the *nef* stop codon to a TGT cysteine codon. The PCR products were subsequently purified and cloned into the pN1 expression vector (Clontech) containing the $3^{\prime }$  *egfp* ORF. To exogenously express SERINC5 *in trans*, a pBJ5-SERINC5.intHA vector was utilized, which was a kind gift from Heinrich Göttlinger (UMass Medical School). This SERINC5 harbors an HA tag inserted between residues 290 and 291 of SERINC5, which is located on the fourth extracellular loop, connecting the seventh and eighth transmembrane helices. Sanger sequencing was performed to confirm the identity of the *nef* and *serinc5* genes.

### Cell-surface CD4 and SERINC5 Downregulation Assay

To determine the ability of each Nef isolate to downregulate cell-surface CD4 and SERINC5 in CD4${}^{+}$ HeLa cells, $2.5\times 10^{5}$ CD4${}^{+}$ HeLa cells were plated in 12-well plates 24 h prior to transfection. The CD4${}^{+}$ HeLa cells were then cotransfected with 0.4 μg of the respective pN1-Nef-eGFP plasmids along with 0.5 μg of the pBJ5-SERINC5.intHA plasmid using PolyJet (FroggaBio, North York, ON, Canada). For cell-surface staining of transfected CD4${}^{+}$ HeLa cells, 24 h post-transfection cells were washed twice with 1× PBS (Wisent), displaced with 0.25% Trypsin (Life Technologies, Carlsbad, CA) and collected into round bottom 96-well plates. Cells were then spun at 500× g for 5 min at room temperature and were subsequently washed twice more with 1× PBS. Pelleted cells were fixed with 1.5% paraformaldehyde (PFA) for 20 min in the dark and washed twice with 200 μl FACS Buffer [3% FBS, 5 mM ethylenediaminetetraacetic acid {EDTA} in 1× PBS]. Cells were subsequently stained with the appropriate antibodies for 60 min while rocking, at room temperature, washed twice with FACS Buffer and resuspended in 1× PBS.

For staining cell surface CD4 and SERINC5, 1:100 PE-conjugated antihuman CD4 antibody (clone OKT4; BioLegend) and 1:500 AlexaFluor647-conjugated anti-HA.11 epitope tag antibody (clone 16B12; BioLegend) in FACS Buffer were used. The isotype control sample in these experiments was stained with 1:50 AlexaFluor647-conjugated mouse IgG1, κ-Isotype Control Antibody (clone MOPC-11; BioLegend) and 1:200 PE-conjugated mouse IgG2B, κ-Isotype Control Antibody (clone MPC-11; BioLegend) in FACS Buffer.

For flow cytometry analysis, cells were analyzed using a BD Biosciences LSR II RRI 4260 flow cytometer (London Regional Flow Cytometry Facility, London, ON, Canada). A Fluorescence Minus One (FMO) control lacking eGFP fluorescence was used to determine the transfected (eGFP${}^{+}$) cell population. The geometric mean fluorescence intensity (gMFI) was then determined for AlexaFluor647 (cell-surface SERINC5 expression), PE (cell-surface CD4 expression), and eGFP (Nef-eGFP expression) within the transfected eGFP${}^{+}$ population for each sample. The fold-SERINC5 and CD4 downregulation ability was determined by comparing the gMFI of AlexaFluor647 and PE in cells transfected with the eGFP-only negative control to cells expressing the respective Nef-eGFP isolates. Fold Nef-eGFP expression was calculated by comparing the gMFI of eGFP in transfected cells expressing the Nef-eGFP isolates to the gMFI of eGFP within cells expressing the eGFP-only negative control, as described (Mumby et al. 2021). Flow cytometry data were analyzed using FlowJo software (version 10.7.1, FlowJo LLC, Ashland, OR).

### Fusion Protein Analysis

To determine the in vitro expression level of all tested Nef-eGFP fusion proteins, in the presence or absence of SERINC5, $5\times 10^{5}$ CD4${}^{+}$ HeLa cells were seeded in 6-well plates, 24 h prior to transfection. To test Nef-eGFP expression, the CD4${}^{+}$ HeLa cells were cotransfected with 0.64 μg of the respective pN1 Nef-eGFP constructs in the absence or presence of 0.80 μg of pBJ5 SERINC5.intHA using PolyJet, as described previously (Mumby et al. 2021). Twenty-four hours post-transfection, the cells were washed once with 1× PBS then lysed by rocking in lysis buffer [0.5 M HEPES, 1.25 M NaCL, 1 M MgCl${}_{2}$, 0.25 M EDTA, 0.1% Triton X-100, and 1× complete Protease Inhibitor Tablets {Roche, Indianapolis, IN}] for 1 h at 4 ${}^{\circ }$C. The supernatant was then clarified by spinning at 20,000× g for 30 min at 4 ${}^{\circ }$C, mixed with SDS-PAGE sample buffer (0.312 M Tris pH 6.8, 3.6 M 2-Mercaptoethanol, 50% glycerol, 10% SDS) and boiled at 98 ${}^{\circ }$C for 8 min. Samples were run on 12% SDS-PAGE gels, followed by transferring to nitrocellulose membranes. Membranes were blocked with 5% milk in TBST (50 mM Tris, 150 mM NaCl, 0.1% Tween 20) for 1 h at room temperature, followed by incubation overnight at 4 ${}^{\circ }$C with the appropriate primary antibody in 5% milk in TBST. The following primary antibodies were used: 1:5,000 mouse anti-GFP monoclonal antibody (ThermoFisher; cat. no.: MA5-15256), 1:20,000 mouse anti-GAPDH monoclonal antibody (ThermoFisher; cat. no.: AM4300). The next day, membranes were washed three times with TBST and incubated for 2 h at room temperature with 1:10,000 (for GAPDH detection) and 1:2,000 (Nef-eGFP detection) of the HRP-conjugated goat antimouse IgG (H + L) secondary antibody (ThermoFisher; cat. no.: 31430) in 5% milk in TBST. Blots were subsequently washed in TBST and developed using Immobilon Classico Western HRP Substrate (for GAPDH detection—Millipore; cat. no.: WBLUC0500), or Immobilon Crescendo Western HRP Substrate (for Nef-eGFP detection—Millipore; cat. no.: WBLUR0500), and imaged using a C-DiGit chemiluminescence Western blot scanner (LI-COR Biosciences, Lincoln, NE).

### Statistical Analysis

We used R version 3.6.3 to perform statistical analysis. We used one-way ANOVA to test for significant variation among groups, and used Tukey’s HSD test to identify significant differences between specific pairs of groups while adjusting for multiple comparisons.

## Results

### Variability of Extant Nef Proteins

Although Nef plays a major role in the infectivity of HIV and SIV, the specific mechanism Nef uses to facilitate a particular function may differ in those derived from HIV-1 and SIV (Collette et al. 2000). We first sought to reconstruct the emergence of the *nef* gene in the evolutionary history of primate lentiviruses, which allowed for functional characterization of the reconstructed proteins leading up to the common ancestor of HIV-1/M in subsequent experiments. We obtained n=34 reported SIV and HIV Nef protein sequences and reconstructed a maximum-likelihood tree. The best-supported substitution model (Nguyen et al. 2015) was the variable time matrix (Müller and Vingron 2000), with empirical frequencies and three nonparametric rate categories. At the protein structural level, HIV-1 Nef is characterized by an intrinsically disordered anchor region at the amino terminus, a folded core region with a flexible internal loop and a C-terminal region (Arold and Baur 2001; Staudt et al. 2020). The flexibility of the unstructured anchor region and internal loop enables the structured core region to form novel protein to protein interactions (Arold and Baur 2001). Among the extant Nef sequences, the sequence length and the predicted intrinsic disorder varies substantially, consistent with the PLV phylogeny (supplementary fig. S2, Supplementary Material online). Thus, the structural and functional variations observed amongst extant PLV Nef proteins may be associated with genetic changes in their shared evolutionary history.

### Ancestral Reconstruction

We used BEAST to generate a posterior sample of time-scaled phylogenies relating Nef sequences. The prior distribution on the time to the most recent common ancestor (TMRCA) was based on a recent phylogenetic study of primate lentiviruses, where the isolation of Bioko drills from the African continent by rising sea levels about 10,000 years before present provided a more accurate estimate of the TMRCA (Worobey et al. 2010). In the present study, we incorporated additional sequences representing SIV, HIV-1 Groups M, N, O, and P. When representing the maximum clade credibility (MCC) tree for this posterior sample, we observed that the internal node representing the common ancestor of all HIV-1/M samples is substantially further back in time than previously described (fig. 3) (Bletsa et al. 2019). This discrepancy was also reported by Worobey et al. (2010), who attributed this to an accelerated rate of evolution in branches leading up to these viruses, but did not rule out the possibility of an earlier origin of HIV-1 lineages. Based on our posterior sample, the most recent common ancestor of PLV Nef emerged about 74.4 thousand years ago (kya; 95% highest probability density 40.92–113.57 kya).

From this posterior sample of trees, we determined the most frequent trajectory of internal nodes from the root to the common ancestor of HIV-1/M. This “maximum credibility” trajectory comprising six nodes (table 1) was found in about 11% of the 4,000 trees in our posterior sample. We extracted a random subsample of 1,000 trees containing this trajectory. Next, we used Historian to reconstruct ancestral Nef protein sequences at the internal nodes of each tree, and then generated consensus sequences across all 1,000 trees.

**Table 1.** Nodes Comprising the Most Common Path from the Root to the HIV-1 Group M (HIV-1/M) Ancestor in the Maximum-Likelihood Tree.

Node	Ancestral Label	Description
35	PLV	All primate lentiviruses including: SIVcpzpts, SIVcpzptt, SIVrcm, SIVsun, SIVgsn/mus/mon HIV-1 (M, N, O, P), and HIV-2
51	HIV-1/SIVsun	HIV-1 Groups M, N, O, P, SIVcpzpts, SIVcpzptt, and SIVsun
52	HIV-1/SIVcpz	HIV-1 Groups M, N, O, P, SIVcpzpts, and SIVcpzptt
55	HIV-1/SIVcpzptt	HIV-1 Groups M, N, O, P, and SIVcpzptt
56	HIV-1/M,N/SIVcpzptt	HIV-1 Groups M, N, and SIVcpzptt
59	HIV-1/M	HIV-1 Group M

Note—Node indices correspond to the default numbering of internal nodes in the R package *ape*, which indexes the tips (n=34) before starting at the root. “Ancestral label” corresponds to the more informative labels of these nodes that we use in this manuscript. Label assignment to sequences in ancestral nodes.

To evaluate the accuracy of this reconstruction method, we simulated sequence evolution under varying evolutionary model parameters, given a fixed sequence at the root of the tree for all 1,000 trees. We measured the error rate by the relative number of nucleotide differences between the reconstructed and actual root sequences. These simulation results are summarized in supplementary figure S3, Supplementary Material online. Overall, the simulated distribution of indel lengths had no substantial effect on these error rates. Error rates increased with the expected number of substitutions in the tree per codon (scaling factor, SF). In other words, increasing genetic variation made it more difficult to reconstruct the root sequence, which was expected. To interpret these results, we calculated the mean amino acid entropies of the observed and simulated sequences. Based on the observed mean entropy (0.246), an SF value between 2.5 and 5 was most consistent with the actual data, which corresponded to an expected error rate of about 0.15 (supplementary fig. S3, Supplementary Material online).

Each consensus Nef sequence (supplementary fig. S4, Supplementary Material online) associated with the six internal nodes on the path from the PLV ancestor to the common ancestor of HIV-1/M was synthesized to determine if these proteins could be expressed in cellulo, and if so, whether these proteins could perform two defined Nef functions: the downregulation of SERINC5 and CD4 from the cell surface. The nomenclature for all nodes are detailed in table 1, and henceforth all Nef proteins will be referred by their ancestral node labels.

### Ancestral Primate Lentivirus (PLV) Nef Robustly Downregulates Cell-Surface SERINC5, but Only Modestly Downregulates Cell-Surface CD4

**Fig. 1.**
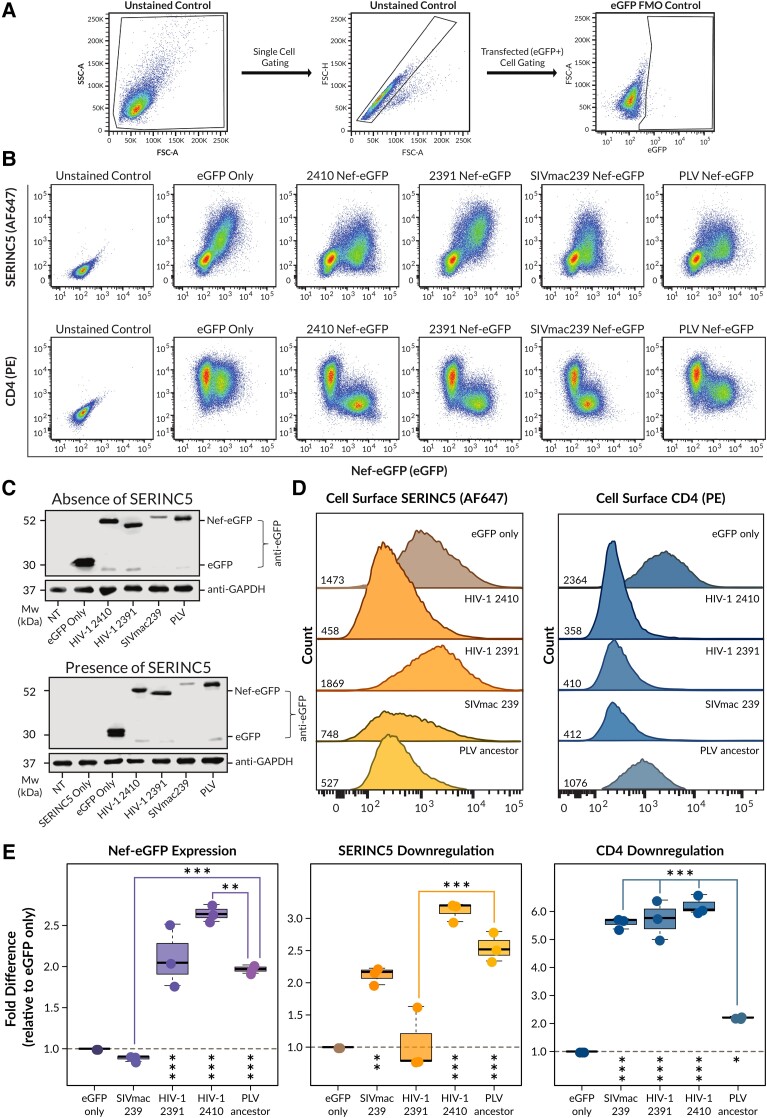
The ancestral primate lentivirus (PLV) Nef protein potently downregulates cell-surface SERINC5, but only modestly downregulates cell-surface CD4. CD4${}^{+}$ HeLa cells coexpressing C-terminally eGFP-tagged ancestral PLV Nef, SIVmac239 Nef or the 2410 and 2391 primary HIV-1 Nef proteins with SERINC5.intHA were stained for cell-surface human SERINC5 and human CD4 and levels were quantified by flow cytometry. Single transfected CD4${}^{+}$ HeLa cells were determined by gating on eGFP${}^{+}$ cells using a fluorescence minus one (FMO) control. (*A*) Schematic of the gating strategy used to determine the transfected (eGFP${}^{+}$) cell population. (*B*) Representative pseudocolor plots illustrating cell-surface SERINC5 (AlexaFluor647; upper panel) and CD4 (PE; lower panel) levels of single cell populations. (*C*) Representative Western blots illustrating the expression of Nef-eGFP fusion proteins in the absence (upper panel) and presence (lower panel) of SERINC5 in CD4${}^{+}$ HeLa cells. (*D*) Representative histograms illustrating cell-surface SERINC5 and CD4 levels on CD4${}^{+}$ HeLa cells after gating on single and eGFP${}^{+}$ cells. Geometric MFIs of the cell-surface proteins are indicated on the *x* axis. (*E*) Summary of the fold Nef-eGFP expression (±SE) and the fold downregulation ability (±SE) for cell-surface SERINC5, and cell-surface CD4 from three independent experiments (n=3). Note—eGFP, enhanced green fluorescent protein; AF647, AlexaFluor647; PE, phycoerythrin; SE, standard error; MFI, mean fluorescence intensity; Mw, molecular weight; NT, nontransfected; kDa, kilodaltons; GAPDH, glyceraldehyde 3-phopshate dehydrogenase. Asterisks under dotted line: Significance fold difference with respect to eGFP only. Asterisks on top of boxplots: Significance fold difference with respect to PLV ancestor. Significance codes: P<0.001 “***”, P<0.01 “**”, P<0.05 “*” (adjusted *P* values reported from Tukey HSD).

To compliment the reconstruction of the ancestral PLV *nef* sequence, we next evaluated if the PLV Nef protein was functional in cellulo by testing cell-surface CD4 and SERINC5 downregulation. A previously described flow cytometry assay was used to measure cell-surface levels of SERINC5 and CD4 in CD4${}^{+}$ HeLa cells coexpressing the PLV Nef protein C-terminally fused to the eGFP fluorophore, and SERINC5 (Mumby et al. 2021). Within these experiments, the ability of two primary HIV-1 Nef proteins, termed 2410 and 2391 Nef, were included (Mumby et al. 2021). We previously defined how these two isolates can robustly downregulate cell-surface CD4, yet only isolate 2410 Nef additionally downregulates SERINC5 from the cell surface efficiently (Mumby et al. 2021). The functional ability of the full-length Nef protein derived from SIVmac239 was also included in these experiments, as this SIV Nef protein can downregulate both SERINC5 (Janaka et al. 2021) and CD4 from the cell surface efficiently (Manrique et al. 2017; Janaka et al. 2021). Indeed, upon comparing to the eGFP-only negative control, the ancestral PLV Nef protein demonstrated an approximate 2.5-fold significant increase in cell-surface SERINC5 downregulation (P<0.001; fig. 1*B*, *D*, *E*). The ability of ancestral PLV Nef to downregulate cell-surface SERINC5 was similar to the abilities of SIVmac239 Nef and 2410 Nef, which displayed an approximate 2-fold (P<0.01) and 3-fold (P<0.001) increase in cell-surface SERINC5 downregulation, respectively (fig. 1*B*, *D*, *E*). Moreover, no significant differences in SERINC5 downregulation were observed between ancestral PLV Nef and 2410 Nef/SIVmac239 Nef, suggesting ancestral PLV Nef can robustly downregulate cell-surface SERINC5. Furthermore, 2391 Nef demonstrated minimal SERINC5 downregulation ability as expected (Mumby et al. 2021) (P>0.05; fig. 1*B*, *D*, *E*).

Next, to determine if ancestral PLV Nef could additionally downregulate cell-surface CD4, we compared cell-surface CD4 levels in cells expressing ancestral PLV Nef to cells expressing eGFP only. As such, ancestral PLV Nef exhibited an approximate 2-fold increase in CD4 downregulation (P<0.05; fig. 1*B*, *D*, *E*), suggesting ancestral PLV Nef can downregulate cell-surface CD4 to some extent. However, ancestral PLV Nef is approximately 3-fold less efficient in reducing cell-surface CD4 upon comparison to 2410, 2391, and SIVmac239 Nef (P<0.001; fig. 1*B*, *D*, *E*), all of which are known to downregulate cell-surface CD4 efficiently (Manrique et al. 2017; Janaka et al. 2021; Mumby et al. 2021). Overall, this suggests ancestral PLV Nef downregulates CD4 from the cell surface modestly. We next calculated the relative expression level of the tested Nef-eGFP fusion proteins. We determined that the ancestral PLV Nef-eGFP protein is expressed abundantly, as evidenced by a 2-fold significant increase in expression compared to cells expressing eGFP only, or SIVmac239 Nef (P<0.001; fig. 1*E*). The expression level of ancestral PLV Nef was comparable to 2391 Nef (P>0.05), but was significantly lower compared to 2410 Nef (P<0.01; fig. 1*E*). Importantly, the trends observed in Nef-eGFP expression via flow cytometry were consistent with the intensity of Nef-eGFP fusion protein band expression as determined via Western blot, both in the absence and presence of SERINC5 (fig. 1*C* and *E*). Furthermore, despite low expression levels, SIVmac239 Nef retained cell-surface SERINC5 and CD4 downregulation (fig. 1*B*–*E*). The Western blot analysis in figure 1*C* revealed the presence of lower molecular weight anti-eGFP reactive proteins that may represent Nef-eGFP degradation products, eGFP on its own, or nonspecific background bands. As such, even though a minor proportion of the signal detected via flow cytometry (fig. 1*E*) may be originating from these lower molecular weight proteins, their presence does not affect our interpretation of Nef functions. Taken together, the ancestral PLV Nef protein can efficiently downregulate cell-surface SERINC5, yet can only partially downregulate cell-surface CD4.

### Cell-Surface CD4 and SERINC5 Capabilities Evolved at Different Times

**Fig. 2.**
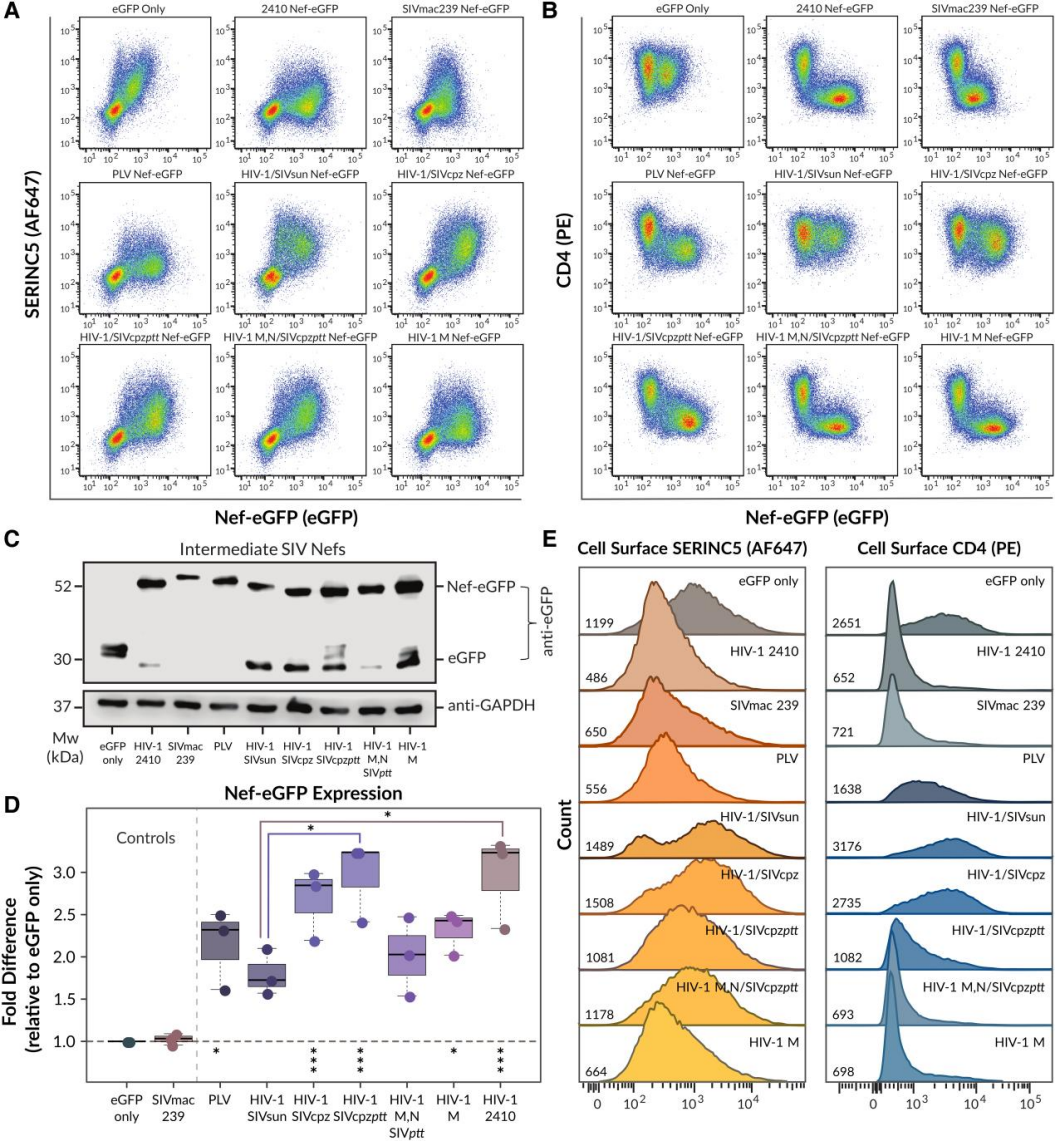
Effect of ancestral Nef proteins on cell-surface SERINC5 and CD4 levels. CD4${}^{+}$ HeLa cells coexpressing the PLV Nef-eGFP protein, SIVmac239 Nef-eGFP, 2410 Nef-eGFP or intermediary nodes 51 (HIV-1/SIVsun), 52 (HIV-1/SIVcpz), 55 (HIV-1/SIVcpzptt), 56 (HIV-1/M, N/SIVcpzptt), and 59 (HIV-1/M) Nef-eGFP with SERINC5.intHA were stained for cell-surface human SERINC5 and human CD4. Subsequent cell-surface levels of SERINC5 and CD4 were then determined using flow cytometry. Single transfected CD4${}^{+}$ HeLa cells were determined by gating on eGFP${}^{+}$ cells using an FMO control. (*A*) and (*B*) Representative pseudocolor plots illustrating cell-surface SERINC5 (AlexaFluor647) and CD4 (PE) levels of single cell populations. (*C*) Representative Western blot illustrating the expression of Nef-eGFP fusion proteins in the presence of SERINC5 in CD4${}^{+}$ HeLa cells. (*D*) Fold Nef-eGFP expression (±SE) in CD4${}^{+}$ HeLa cells (n=3). (*E*) Representative histograms illustrating cell-surface SERINC5 and CD4 levels on CD4${}^{+}$ HeLa cells after gating on single, eGFP${}^{+}$ cells. Geometric MFIs of the cell-surface proteins are indicated on the *x* axis. Note—eGFP, enhanced green fluorescent protein; AF647, AlexaFluor647; PE, phycoerythrin; SE, standard error; FMO, fluroescence minus one; MFI, mean fluorescence intensity; Mw, molecular weight; kDa, kilodalton; GAPDH, glyceraldehyde 3-phosphate dehydrogenase. Asterisks under dotted line: Significance fold difference with respect to eGFP only. Asterisks on top of boxplots: Significance fold difference among adjacent groups. Significance codes: P<0.001 “***”, P<0.01 “**”, P<0.05 “*” (adjusted *P* values reported from Tukey HSD).

**Fig. 3.**
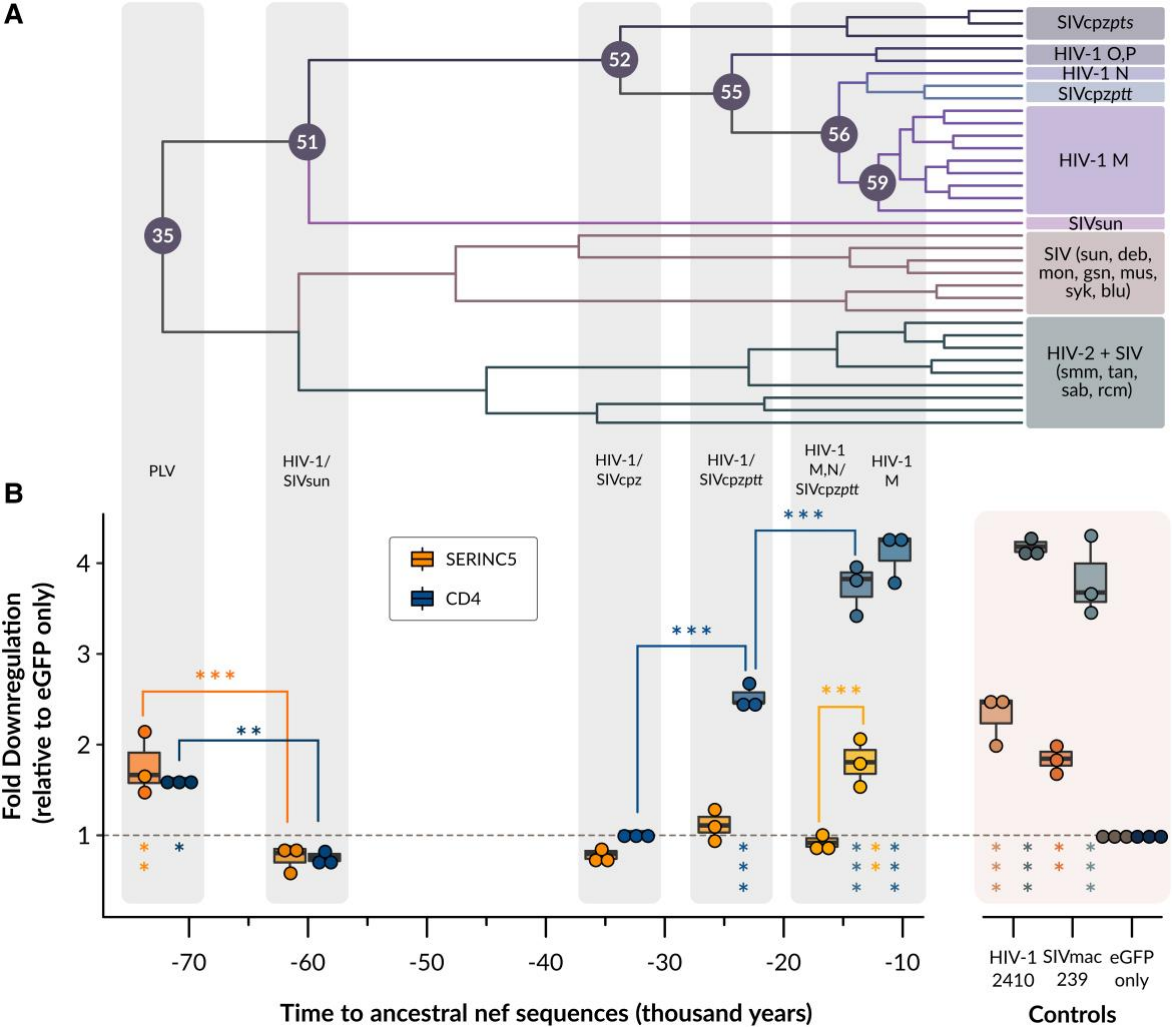
Predicting the evolution of Nef-mediated SERINC5 and CD4 cell-surface downregulation. (*A*) Maximum clade credibility tree (MCC) of 1,000 BEAST trees with consistent nodes used to obtain ancestral Nef sequences. (*B*) Fold downregulation ability (±SE) for cell-surface SERINC5 (orange), and cell-surface CD4 (blue) of the Nef ancestral proteins from the root (PLV Nef) to the tip (HIV-1/M Nef). Note—eGFP, enhanced green fluorescent protein. Asterisks under dotted line: Significance fold difference with respect to eGFP only. Asterisks on top of boxplots: Significance fold difference among adjacent groups. Significance codes: P<0.001 “***”, P<0.01 “**”, P<0.05 “*” (adjusted *P* values reported from Tukey HSD).

Given that the PLV Nef protein can perform key Nef functions such as cell-surface SERINC5 and CD4 downregulation, we next sought to determine whether these functions were preserved along the evolutionary lineage from the PLV ancestor to modern-day HIV-1/M. Cells coexpressing either ancestral PLV Nef-eGFP, SIVmac239Nef-eGFP, 2410 Nef-eGFP and Nef from intermediary nodes 51 (HIV-1/SIVsun, table 1), 52 (HIV-1/SIVcpz, table 1), 55 (HIV-1/SIVcpzptt, table 1), 56 (HIV-1/M, N/SIVcpzptt, table 1), 59 (HIV-1/M, table 1) Nef-eGFP, in addition to SERINC5.intHA, were stained for surface human SERINC5 and human CD4. We observed that the HIV-1/SIVsun ancestor, the most distant ancestor of the HIV-1/M lineage apart from the root, suffered major impairments in cell-surface SERINC5 downregulation (P>0.05; figs. 2*A*, 2*E*, 3*A*, and 3*B*). This inability to downregulate cell-surface SERINC5 persisted up to and including the HIV-1/M,N/SIVcpzptt ancestor (Node 56; figs. 2*A*, 2*E*, 3*A*, and 3*B*). Accordingly, the HIV-1/M ancestral (Node 59) Nef protein demonstrated a robust, approximate 1.8-fold increase in cell surface SERINC5 downregulation (P<0.01), equivalent to the extent that ancestral PLV Nef downregulates SERINC5 (P<0.01; figs. 2*A*, 2*E*, 3*A*, and 3*B*). This is striking, as the branch length between the HIV-1/M,N/SIVcpzptt ancestor (Node 56) and the HIV-1/M ancestor (Node 59) is relatively short, implying that these changes accumulated over a relatively short period of time (fig. 3*B*).

In parallel, we also assessed cell-surface CD4 downregulation in the presence of the intermediary Nodal Nef-eGFP fusion proteins. In these experiments, ancestral PLV Nef once again demonstrated a minor, yet significant 1.5-fold increase in cell-surface CD4 downregulation upon comparison to cells expression eGFP only (P<0.05; figs. 2*B*, 2*E*, 3*A*, and 3*B*). Despite exhibiting only a modest capability to downregulate cell-surface CD4, ancestral PLV Nef downregulated cell-surface CD4 more efficiently than the HIV-1/SIVsun Nef ancestor (Node 51; P<0.01; figs. 2*B*, 2*E*, 3*A*, and 3*B*), which itself did not differ in this functional ability compared to the HIV-1/SIVcpz ancestor (Node 52; P>0.05; figs. 2*B*, 2*E*, 3*A*, and 3*B*). This suggests both the HIV-1/SIVsun Nef and HIV-1/SIVcpz Nef ancestors are deficient in their respective abilities to downregulate cell-surface CD4. Moreover, while the HIV-1/SIVsun Nef ancestor (Node 51) and the HIV-1/SIVcpz Nef ancestor (Node 52) were unable to downregulate cell-surface CD4, the HIV-1/SIVcpzptt Nef ancestor (Node 55) rapidly regained this functional ability (figs. 2*B*, 2*E*, 3*A*, and 3*B*). Indeed, the HIV-1/SIVcpzptt Nef ancestor (Node 55) demonstrated a 2.5-fold increase in cell-surface CD4 downregulation (P<0.001), which was significantly greater to cells expressing the ancestral PLV Nef protein (P<0.001; figs. 2*B*, 2*E*, 3*A*, and 3*B*). Furthermore, the HIV-1/M,N/SIVcpzptt Nef ancestor (Node 56) and the HIV-1/M Nef ancestor (Node 59) both demonstrated an approximate 4-fold increase in cell-surface CD4 downregulation (P<0.001), which was significantly greater than CD4 downregulation by the HIV-1/SIVcpzptt Nef ancestor (Node 55; P<0.001) and similar to SIVmac239 Nef and 2410 Nef CD4 downregulation levels (figs. 2*B*, 2*E*, 3*A*, and 3*B*). While the level of eGFP expression as determined by flow cytometry was variable between cells expressing the intermediary Nodal Nef-eGFP fusion proteins (table 1), these differences were not significant, with the exception of the HIV-1/SIVcpzptt ancestor being expressed significantly higher than the HIV-1/SIVsun ancestor (P<0.05) (fig. 2*D*). To ascertain this, we also compared Nef-eGFP fusion protein levels using Western blot analysis and determined that the Nef-eGFP fusion protein band intensities were consistent with the trends in eGFP expression levels as determined via flow cytometry (fig. 2*C* and *D*). Thus, given that ancestral PLV Nef could downregulate cell-surface SERINC5 and CD4, but the HIV-1/SIVsun Nef ancestor failed to do so, these results suggest that both of these Nef functions were lost in the early stages of SIVcpz evolution to chimpanzees. While the ability of Nef to downregulate CD4 gradually evolved over time, the finding that the HIV-1/M ancestral Nef protein was able to efficiently downregulate SERINC5, which was lost in all preceding ancestral nodes, suggests this function could be rapidly re-acquired given the right selective pressures.

## Discussion

Ancestral protein sequence reconstruction methods allow an estimation of sequences at different time points in a phylogeny. Downstream, these sequences could be resurrected in the laboratory using experimental techniques, or analyzed computationally to characterize the evolution of genes, proteins, or genomes (Chang and Donoghue 2000). Here, we reconstructed Nef protein sequences in the primate lentivirus phylogeny and characterized these sequences using both computational and experimental techniques.

Our functional analysis primarily characterized the ability of ancestral SIV Nef to downregulate cell-surface human SERINC5 and human CD4. We assessed if these functional outputs were consistent with prior observations of SIV Nef protein function, and evaluated the accuracy of the ancestral *nef* reconstruction protocol herein. Our results suggested that ancestral PLV Nef robustly downregulated cell-surface SERINC5, yet only modestly downregulated cell-surface CD4. In the context of SERINC5 downregulation, this is consistent with prior observations demonstrating that Nef proteins derived from SIV capable of infecting Old-World monkeys can often efficiently antagonize SERINC5 in a species-independent manner (Heigele et al. 2016). Moreover, the finding that SIVmac239 Nef efficiently downregulated cell-surface SERINC5 (figs. 1 and 2) is in agreement with prior reports that SIVmac239 Nef is a potent antagonist of SERINC5 (Janaka et al. 2021; Heigele et al. 2016). Interestingly, prior studies have demonstrated that while Nef proteins derived from modern-day SIVcpzpts and SIVcpzptt can generally antagonize SERINC5 potently, Nef proteins derived from the SIVrcm and SIVmus/mon/gsn lineages, the precursors of SIVcpz, exhibited poor SERINC5 antagonism (Heigele et al. 2016). In the present study, we only assessed the ability of the reconstructed ancestral Nef proteins to downregulate SERINC5 and CD4 from the cell surface. While Nef-mediated SERINC5 downregulation may represent the key mechanism of SERINC5 antagonism to restore virion infectivity (Rosa et al. 2015), it remains unknown whether the ability of reconstructed Nef proteins to downregulate cell-surface SERINC5 rescues virion infectivity. Furthermore, the contribution of SERINC3 to virion infectivity (Usami et al. 2015) in the presence of the ancestral Nef proteins is also unknown. Indeed, naturally occurring HIV-1 Nef polymorphisms downregulate SERINC3 and SERINC5 differentially, suggesting SERINC5 downregulation may not necessarily indicate efficient SERINC3 downregulation (Jin et al. 2020). Moreover, HIV-1 Env can also confer resistance to SERINC5 restriction, and this is independent of Nef (Beitari et al. 2017). As such, the role of Nef and Env, as well as any potential functional interplay, could have been under equivalent or differential selective pressures. Indeed, SERINC5 incorporation into virions can stabilize an open conformation of both SERINC5-sensitive and SERINC5-resistant Env, which exposes regions of Env to recognition by some neutralizing antibodies (Beitari et al. 2017; Sood et al. 2017; Kirschman et al. 2022).

Our findings suggest that Nef-mediated SERINC5 downregulation was lost from ancestral PLV Nef to the SIVrcm and SIVmus/mon/gsn lineages, where certain mutations enhancing SERINC5 cell-surface downregulation were selected for during viral adaptation of SIVcpz within the new chimpanzee hosts. We recognize that the root PLV sequence is the most difficult to reconstruct (with an estimated mean error of about 15%, supplementary fig. S3, Supplementary Material online), so the following interpretations are subject to this caveat. Our analysis of SERINC5 downregulation in the intermediate nodal Nefs revealed that SERINC5 downregulation was completely lost in the HIV-1/SIVsun ancestor (Node 51 Nef), the first node of the SIVcpz lineage within our model system. This function was only recovered in the HIV-1/M ancestor (Node 59 Nef), the most distant node of the SIVcpz lineage and ancestor of HIV-1/M Nef, where SERINC5 downregulation was restored to levels similar to ancestral PLV Nef. This supports prior claims that SERINC5 antagonism was lost in the SIVrcm and SIVmus/mon/gsn lineages prior to the establishment of SIVcpz within chimpanzees (Heigele et al. 2016). While this may not directly explain how Nef derived from SIVcpzpts and SIVcpzptt evolved to efficiently antagonize SERINC5 as the HIV-1/SIVsun (Node 51), HIV-1/SIVcpz (Node 52), HIV-1/SIVcpzptt (Node 55) and the HIV-1/M,N/SIVcpzptt (Node 56) Nef ancestors failed to downregulate SERINC5 altogether (fig. 2), the sudden emergence of SERINC5 downregulation within the HIV-1/M ancestor (Node 59 Nef) may serve as a proof-of-concept that this function could evolve rapidly given the right selective pressures. Indeed, the prevalence of SIV within a given wild host population positively correlates with the extent to which Nef proteins antagonize SERINC5 (Heigele et al. 2016). This suggests that the positive selection of mutations enhancing SERINC5 antagonism are critical for increasing the dissemination of SIV within its respective host population. Given the known negative effects of pervasive SIVcpz infections on afflicted wild chimpanzee populations (Keele et al. 2009; Rudicell et al. 2010), we speculate that early in its evolutionary history, SIVcpz was poorly transmissible as a result of its highly pathogenic nature. As such, this may have created the necessary pressures where increased SERINC5 antagonism would be advantageous, thereby increasing transmissibility within the new chimpanzee hosts.

While it is plausible that SERINC5 antagonism evolved to overcome inefficient transmission events during early SIVcpz adaptation to chimpanzees, it is also conceivable that SERINC5 antagonism coevolved with other Nef functions, namely tetherin downregulation (Buffalo, Stürzel, et al. 2019). While HIV-1 utilizes its Vpu protein to downregulate tetherin (Sauter et al. 2009a; Kmiec et al. 2016; Prévost et al. 2020), most SIVs, including SIVcpz, utilize their respective Nef proteins to antagonize tetherin (Jia et al. 2009; Zhang et al. 2009). In fact, SIVcpz encodes a *vpu* gene derived from SIVmus/mon/gsn, yet its Vpu protein is inactive against chimpanzee tetherin (Sauter et al. 2009b). In the context of SIVcpz adaptation to chimpanzees, a strong selective pressure enabling enhanced Nef-mediated chimpanzee tetherin antagonism would had to have been present (Lim et al. 2010). Indeed, SIVcpz Nef antagonizes chimpanzee tetherin to a greater extent than SIVrcm Nef without losing its ability to antagonize tetherin derived from red-capped mangabeys (Kobayashi et al. 2014). This suggests that SIVcpz Nef underwent gain of function evolution throughout its adaptation to chimpanzees to improve targeting of the new host-specific chimpanzee tetherin restriction factor (Kobayashi et al. 2014). Interestingly, a SIVsmm Nef H${}_{192}$D mutation can disrupt sootey mangabey (*C. atys*) tetherin downregulation and severely affects SERINC5 antagonism without affecting CD4 downregulation (Buffalo, Stürzel, et al. 2019). This suggests that Nef utilizes similar mechanisms to target both tetherin and SERINC5, which are distinct from the mechanisms utilized to downregulate CD4, despite a redundancy in Nef functional motifs utilized (Buffalo, Stürzel, et al. 2019). While we theorize Nef-mediated SERINC5 and tetherin antagonism are closely related functions mechanistically, V${}_{182}$S and H${}_{192}$A mutations induced within SIVmac239 Nef uncouple tetherin and SERINC5 antagonism, suggesting these Nef functions are also distinct (Janaka et al. 2022). As such, we speculate that Nef's ability to antagonize SERINC5 likely coevolved with its ability to downregulate tetherin, which we predict maximizes its fitness advantage by drastically improving the efficiency of such transmission events to a new host species.

The finding that ancestral PLV Nef only modestly downregulated cell-surface human CD4 was unexpected considering the known conservation of this function in multiple SIV and HIV-1 lineages (Benson et al. 1993; Heigele et al. 2016). As we assayed human CD4 downregulation within a human cell line, it is possible ancestral PLV Nef optimally adapted to downregulate its host-specific autologous CD4 protein, which consequently negatively affected its ability to downregulate human CD4. This has been observed in prior studies analyzing the ability of Nef proteins derived from HIV-1, SIVcpz, SIVrcm, and SIVmus/mon/gsn to downregulate both human CD4 and chimpanzee/red-capped mangabey CD4 (Nakano et al. 2015). While the cytoplasmic tails (CTs) of CD4 are generally well conserved amongst primates (Fomsgaard et al. 1992; Nakano et al. 2015), the human CD4 CT differs from chimpanzee/red-capped mangabey CD4 CT only at amino acid position 430, where human CD4 CT and chimpanzee/red-capped mangabey CD4 CT encode a glutamic acid residue (E${}_{430}$) and glutamine residue (Q${}_{430}$), respectively (Nakano et al. 2015). Nef proteins derived from SIVmus/mon/gsn downregulated human CD4 robustly, yet inefficiently downregulated chimpanzee/red-capped mangabey CD4 (Nakano et al. 2015). While the CD4 sequences of the Mona monkey (*C. mona*) and the Greater-Spot Nosed monkey (*C. nictitans*) are not readily available, E${}_{430}$ is also encoded in CD4 from the Mustached Guenon (*C. cephus*; Genbank accession LC017837). This suggests that Nef proteins derived from SIVmus, and perhaps SIVmon and SIVgsn, downregulated human CD4 more efficiently than chimpanzee/red-capped mangabey CD4 due to expression of E${}_{430}$. Furthermore, Nef derived from SIVlho, SIVdeb and SIVagmSAB also inefficiently downregulate human CD4 (Heigele et al. 2016).

SIVcpz Nef downregulates both human CD4 and chimpanzee/red-capped mangabey CD4 more efficiently than SIVrcm Nef, suggesting SIVcpz evolved this enhanced functional capability throughout its adaptation to chimpanzees (Nakano et al. 2015). As it is generally accepted that SIVcpz Nef is derived directly from SIVrcm Nef (Bailes et al. 2003), this suggests that human CD4 and chimpanzee/red-capped mangabey CD4 downregulation was relatively inefficient in the SIVrcm lineage and therefore may have improved once SIVcpz was established in chimpanzees. Our analysis of human CD4 downregulation in the intermediate nodal Nef ancestral proteins revealed that this function was completely lost in the HIV-1/SIVsun ancestor (Node 51 Nef) and the HIV-1/SIVcpz ancestor (Node 52 Nef), recovered partially in the HIV-1/SIVcpzptt ancestor (Node 55 Nef) and eventually fully recovered in the HIV-1/M,N/SIVcpzptt ancestor (Node 56 Nef) and the HIV-1/M ancestor (Node 59 Nef). Unlike SERINC5 downregulation, which evolved rapidly in the HIV-1/M Nef ancestor (Node 59 Nef), our results suggest that human CD4 downregulation evolved gradually throughout SIVcpz adaptation to chimpanzees. While it remains unclear why SIVrcm Nef is unable to downregulate its autologous CD4 receptor efficiently relative to SIVcpz (Nakano et al. 2015), the use of human cell lines may explain some these functional differences. Indeed, due to its lack of enzymatic activity, Nef interacts directly with numerous host cellular proteins to subvert host functions during infection (Johnson et al. 2016; Mumby et al. 2021). Considering that the red-capped mangabey as an Old-World monkey species is more phylogenetically distinct than humans and chimpanzees, certain host factors necessitating a given Nef function in the context of red-capped mangabeys may not be expressed or required for equivalent functions between humans and chimpanzees.

The molecular clock analysis of PLV sequences (Worobey et al. 2010) indicates that ancestral PLV Nef has been circulating in primates for at least 40 thousand years since the common ancestor. Humans have a long history of interacting with wildlife including other primates, and this has led to multiple cross species transmission events of infectious diseases to humans over time (Cunningham et al. 2017). As humans have occasionally encountered primate lentiviruses for much longer than we have estimated, it is possible that behavioral changes were necessary for HIV-1/M to establish as the most successful primate lentivirus in humans (Worobey et al. 2010). In this study, we successfully reconstructed functional PLV Nef proteins associated with the origin of primate lentiviruses as well as intermediate ancestors leading up to HIV-1 group M. We also propose that this Bayesian phylogenetic and molecular cloning workflow provides another approach for resurrecting and studying ancestral proteins (Harms and Thornton 2010).

## Supplementary Material

msad164_Supplementary_Data

## Data Availability

Our study used primate lentiviral *nef* sequences that were published in the NCBI Genbank database under the following accession numbers: AF005494, AF005496, AF061642, AF082395, AF103818, AF382828, AF382829, AF447763, AF468659, AJ006022, AJ249239, AY331295, AY340700, AY340701, AY523865, AY523867, AY536914, AY536915, DQ092760, DQ092762, DQ092764, DQ092766, DQ222472-DQ222476, GU111555, K03454, L20587, M30502, U04005, U46016, and U51190. An alignment of all sequences used in this study, annotated with well-established functional motifs, is provided as supplementary fig. S4. Supplementary data, including the reconstructed ancestral consensus nucleotide sequences generated in this study, are published under the Creative Commons Atribution 4.0 International license at Zenodo: https://doi.org/10.5281/zenodo.8010261.
