# Supplementary material for: Phylogenetic Reconstruction and Functional Characterization of the Ancestral Nef Protein of Primate Lentiviruses
Source: Mol Biol Evol. 2023 Jul 18;40(8):msad164. doi: 10.1093/molbev/msad164 (PMC10400143; doi:10.1093/molbev/msad164)
Supplement: msad164_Supplementary_Data [file msad164_supplementary_data.pdf]

Phylogenetic reconstruction and functional characterization of the ancestral Nef protein of primate lentiviruses

Abayomi S. Olabode[1*], Mitchell J. Mumby[2*], Tristan A. Wild[2], Laura Muñoz Baena[2], Jimmy D. Dikeakos[2], Art FY. Poon[1,2,3]

[1]Department of Pathology & Laboratory Medicine, Western University, London, Canada
[2]Department of Microbiology & Immunology, Western University, London, Canada
[3]Department of Applied Mathematics, Western University, London, Canada
[*]These authors contributed equally to this manuscript

Supplementary Figures

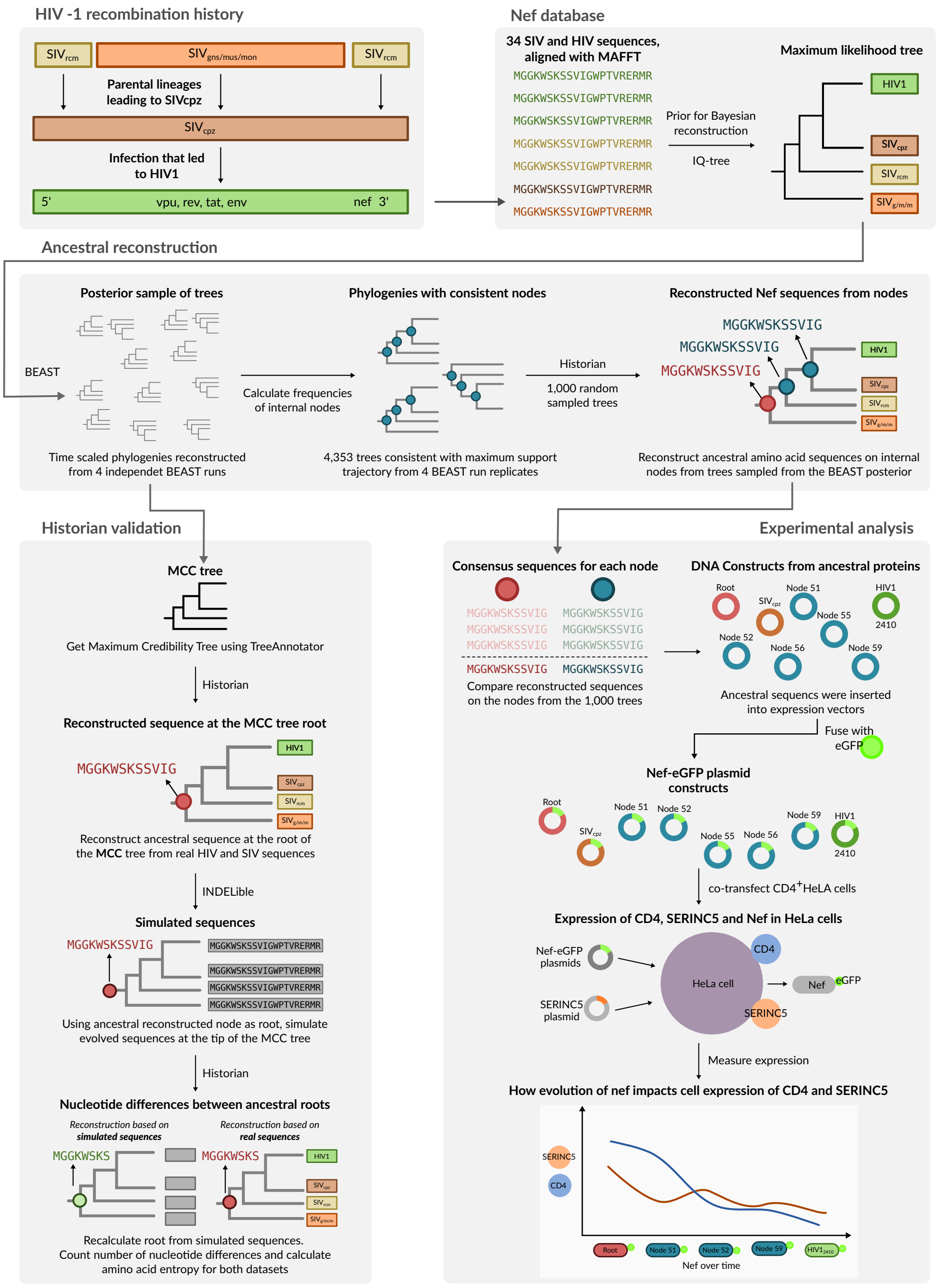

Figure S1: Graphical description of the pipeline used to reconstruct ancestral Nef sequences, and assess the effect of ancestral Nef proteins on cell surface levels of CD4 and SERINC5.

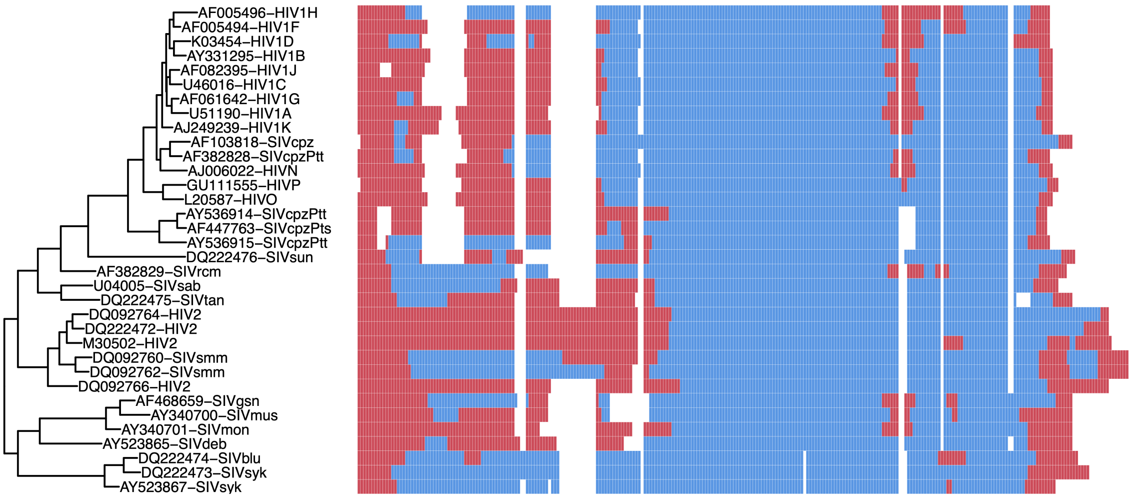

Figure S2: Intrinsic disorder in primate lentivirus Nef proteins. Phylo-heatmap showing the distribution of intrinsic disordered regions in $n = 34$ extant primate lentivirus Nef proteins. Based on previous work [**?**], we used SPOT-Disorder2 [**?**] to detect the presence of intrinsic disorder in the reconstructed Nef sequences. A maximum likelihood tree relating the sequences is displayed on the left. Each tile in the heatmap represents an amino acid, coloured red to indicate that it is predicted to be associated with a region of intrinsic disorder, and blue otherwise. White tiles represent gaps in the aligned amino acid sequences due to indels.

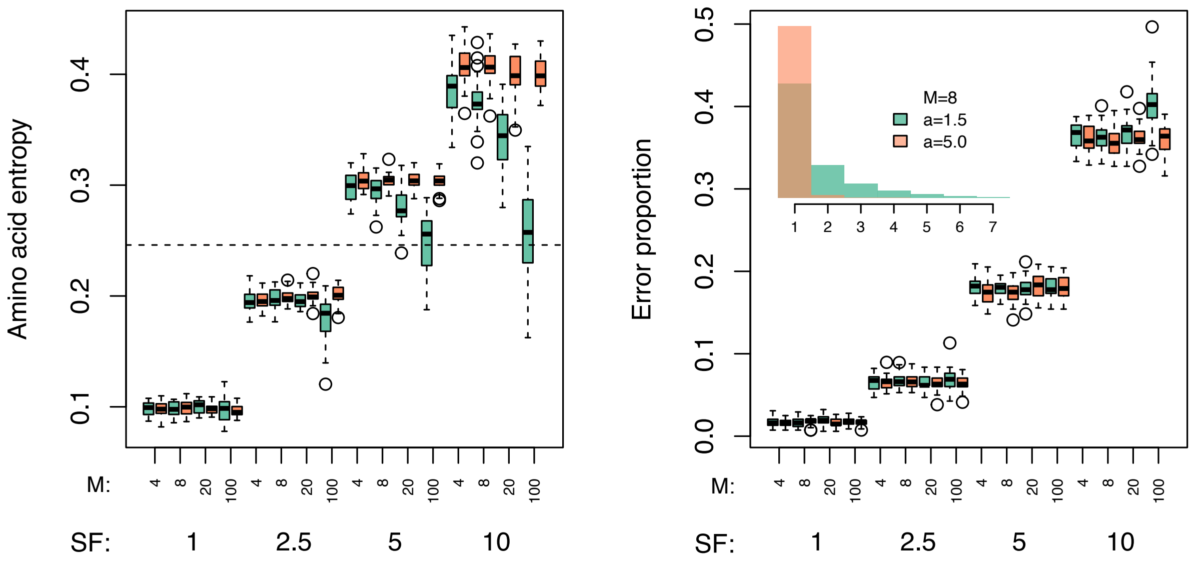

Figure S3: Simulation results. The program INDELible was used to simulate the molecular evolution of *nef* sequences along the maximum credibility tree relating primate lentivirus sequences (Figure **??**A). The amount of genetic variation among simulated sequences was controlled by a scaling factor (SF) that corresponds to the expected number of substitution events per codon in the entire tree. Indel lengths were controlled by the Lavalette distribution, which has two parameters: the maximum indel length, M, and the exponent, a. Examples of this distribution are displayed as an inset figure in the right-hand plot. We quantified the genetic variation by the mean amino acid entropy (*left*). A dashed line indicates the mean entropy for the observed Nef protein sequences (0.246). Next, we measured the number of differences between the nucleotide sequences reconstructed at the root with the program Historian and the true sequence, and normalized by the sequence length to obtain the error proportion (*right*). The number of amino acid differences were highly concordant with the number of nucleotide differences (Lin's concordance correlation coefficient, $\rho_c = 0.967$, 95% C.I. $0.968 - 0.970$).

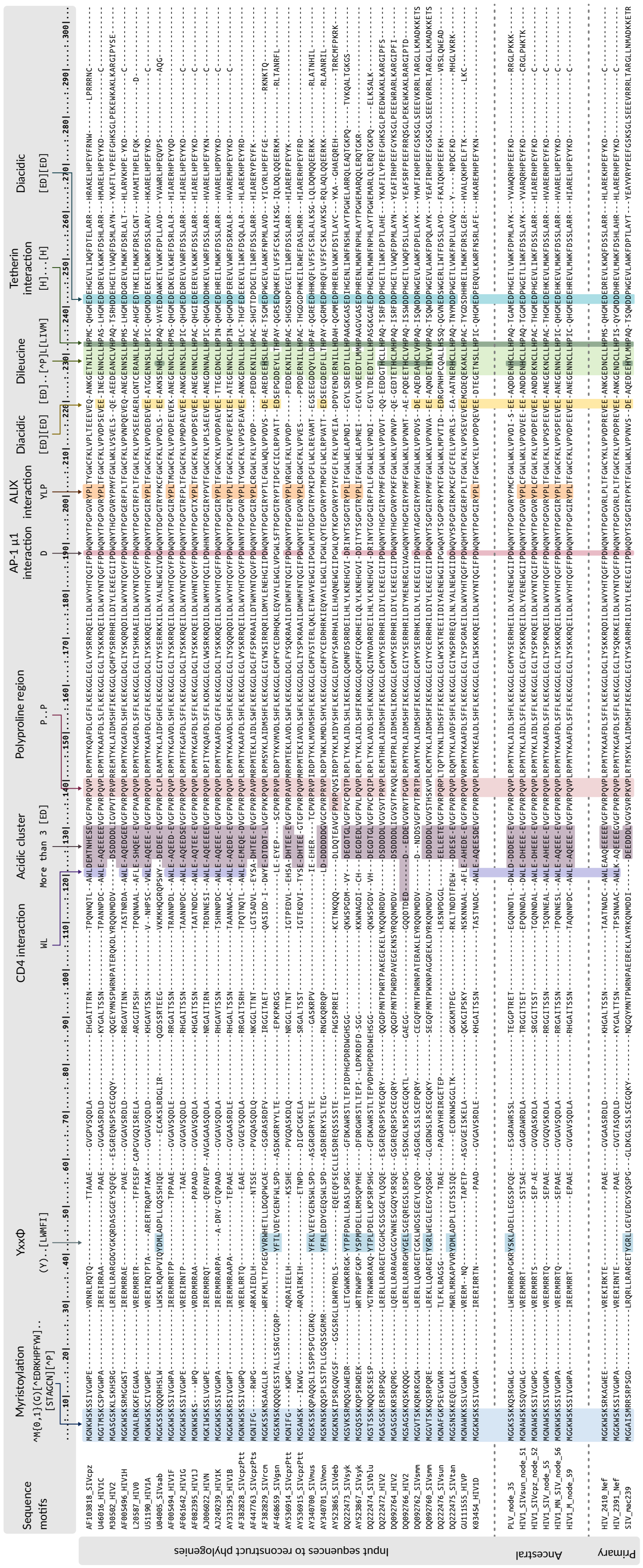
Sequence motifs
Myristoylation
^M{0,1}(G)[^EDRKHPFYW]..[STAGCN][^P]
YxxΦ
(Y)..[LWMFI]
CD4 interaction
WL
Acidic cluster
More than 3 [ED]
Polyproline region
P..P
AP-1 µ1 interaction
D
ALIX interaction
YLP
Diacidic
[ED][ED]
Dileucine
[ED]..[^P]L[LIVM]
Tetherin interaction
[H]...[H]
Diacidic
[ED][ED]
Input sequences to reconstruct phylogenies
Ancestral
Primary

Figure S4: (*previous page*) Alignment of 34 SIV and HIV Nef protein sequences used to reconstruct the Nef phylogeny, and the 9 protein sequences evaluated experimentally (6 reconstructed proteins from ancestral analysis, HIV-1 2410, HIV-1 2391, and SIVmac239 Nef). Selected motifs relevant for Nef's function [**?**] are highlighted in the sequences whenever they are present.
